# Establishment of air kerma reference standard for low dose rate Cs‐137 brachytherapy sources

**DOI:** 10.1120/jacmp.v12i4.3553

**Published:** 2011-11-15

**Authors:** Sunil Dutt Sharma, Sudhir Kumar, P. Srinivasan, G. Chourasiya

**Affiliations:** ^1^ Radiological Physics & Advisory Division Bhabha Atomic Research Centre Anushaktinagar Mumbai 400094 India; ^2^ Radiation Safety Systems Division Bhabha Atomic Research Centre Trombay Mumbai 400085 India

**Keywords:** brachytherapy, ${\;}^{137}\text{Cs}$ source, air kerma, calibration, reference standard

## Abstract

A guarded cylindrical graphite ionization chamber of nominal volume $1000\;{\text{cm}}^{3}$ was designed and fabricated for use as a reference standard for low‐dose rate ${\;}^{137}\text{Cs}$ brachytherapy sources. The air kerma calibration coefficient $\left(N_{K}\right)$ of this ionization chamber was estimated analytically using Burlin's general cavity theory, as well as by the Monte Carlo simulation and validated experimentally using Amersham CDCS‐J‐type ${\;}^{137}\text{Cs}$ reference source. In the analytical method, the $N_{K}$ was calculated for 662 keV gamma rays of ${\;}^{137}\text{Cs}$ brachytherapy source. In the Monte Carlo method, the geometry of the measurement setup and physics‐related input data of the ${\;}^{137}\text{Cs}$ source and the surrounding material were simulated using the Monte Carlo N‐Particle code. The photon energy fluence was used to arrive at the reference air kerma rate (RAKR) using mass energy absorption coefficient. The energy deposition rates were used to simulate the value of charge rate in the ionization chamber, and the $N_{K}$ was determined. The analytical and Monte Carlo values of $N_{K}$ of the cylindrical graphite ionization chamber for ${\;}^{137}\text{Cs}$ brachytherapy source are in agreement within 1.07%. The deviation of analytical and Monte Carlo values from experimental values of $N_{K}$ is 0.36% and 0.72%, respectively. This agreement validates the analytical value, and establishes this chamber as a reference standard for RAKR or AKS measurement of ${\;}^{137}\text{Cs}$ brachytherapy sources.

PACS numbers: 87.53.Bn, 87.53.Jw, 87.56.bg, 87.55.Qr

## I. INTRODUCTION

Sealed gamma rays emitting radioactive sources with average energy higher than 50 keV are extensively used for treatment of various types of cancers employing the technique of brachytherapy.^(^ ^1^ ^)^ Low‐activity (about 3–4 GBq) ${\;}^{137}\text{Cs}$ tube sources of typical diameter 3.0 mm and length 10 to 20 mm are primarily used for low‐dose rate (LDR) treatment of gynecologic cancers through intracavitary implants.^(^ ^1^ ^–^ ^7^ ^)^ Although faster treatment delivery on out‐patient basis through high‐dose rate (HDR) modalities using ${\;}^{192}\text{Ir}$ source is becoming a popular choice in brachytherapy, the LDR treatments using low‐activity ${\;}^{137}\text{Cs}$ source is still thought to be a clinically viable modality in gynecologic brachytherapy.^(^ ^4^ ^,^ ^8^ ^–^ ^9^ ^)^ A wide variety of ${\;}^{137}\text{Cs}$ tube sources are available commercially from different vendors for brachytherapy applications.^(^ ^10^ ^–^ ^14^ ^)^ Even a few new models of these sources have recently been made available for such applications, and their comprehensive dosimetry data have also been published.^(^ ^15^ ^–^ ^17^ ^)^ Recent evaluation of dosimetry for a variety of ${\;}^{137}\text{Cs}$ source designs allows for treatment planning using the current AAPM TG‐43 protocol.^(^ ^18^ ^)^ In India, about 70 radiotherapy centers are using ${\;}^{137}\text{Cs}$ tube sources for manual intracavitary brachytherapy applications.

The measurement of source strength on receipt from the vendor is an important component of brachytherapy quality control because the accuracy of radiation dose delivered to the patient directly depends on the accuracy of source strength measurement.^(^ ^1^ ^,^ ^7^ ^,^ ^14^ ^,^ ^19^ ^–^ ^21^ ^)^ This necessitates the use of a dosimeter which has been calibrated against a reference standard for the radiation source concerned. Measuring the source strength in terms of reference air kerma rate (RAKR) or air kerma strength (AKS) is recommended for gamma‐emitting brachytherapy sources, including low‐activity ${\;}^{137}\text{Cs}$ tube sources used for intracavitary brachytherapy applications.^(^ ^1^ ^,^ ^7^ ^,^ ^14^ ^,^ ^18^ ^–^ ^28^ ^)^ RAKR is the kerma rate to air, in air, at a reference distance of one meter corrected for attenuation and scattering, and refers to the quantity determined along the transverse bisector of the source. AKS is the kerma rate to air, in air, along the transverse bisector of the source at a given distance corrected for attenuation and scattering and multiplied by the square of the distance. Due to low activity and the resulting low signal at a typical calibration distance, it is difficult to measure the RAKR or AKS of ${\;}^{137}\text{Cs}$ brachytherapy sources using small volume ionization chambers. Hence, large volume cylindrical or spherical ionization chambers are selected as reference ionization chambers, due to their high sensitivity and long‐term stability.^(^ ^29^ ^,^ ^30^ ^)^

A guarded cylindrical graphite ionization chamber of nominal sensitive volume $1000\;{\text{cm}}^{3}$ was designed and fabricated locally for strength measurement of low‐activity ${\;}^{137}\text{Cs}$ brachytherapy sources. This newly designed ionization chamber will be maintained as the national RAKR reference standard for LDR Cs‐137 brachytherapy sources and will be used for calibrating the hospital's dosimeters. The air kerma calibration coefficient $\left(N_{K}\right)$ of this ionization chamber was estimated by three independent methods: (i) analytically using Burlin's general cavity theory, (ii) Monte Carlo simulation, and (iii) experimentally using IAEA‐calibrated Amersham CDCS‐J‐type ${\;}^{137}\text{Cs}$ reference source. This paper describes and compares, in detail, the methods of determining $N_{K}$ of this ionization chamber for ${\;}^{137}\text{Cs}$ brachytherapy sources.

## II. MATERIALS AND METHODS

### A. The cylindrical ionization chamber

**Figure 1:**
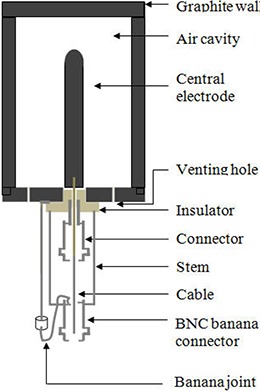
Schematic cross section of cylindrical graphite ionization chamber.

A guarded cylindrical ionization chamber of nominal volume $1000\;{\text{cm}}^{3}$ was fabricated locally using graphite of density $1.76\;g/{\text{cm}}^{3}$. Figure 1 shows the schematic diagram of this ionization chamber. The chamber body was assembled from three graphite components, namely, the cylindrical shell (closed from the top), the central electrode, and the bottom plate. The cylindrical shell has a nominal inner diameter of 10.0 cm and a height of 12.8 cm. The graphite wall of the shell (i.e., chamber) is 4 mm thick, which is sufficient to provide charge particle equilibrium (CPE) for ${\;}^{137}\text{Cs}$ gamma rays. Though nominal wall thickness of 2 mm of graphite is sufficient to establish transient charged particle equilibrium for ${\;}^{137}\text{Cs}$ gamma rays,^(^ ^29^ ^)^ a 4 mm wall thickness was selected due to the machining problems of the graphite. The central electrode of the chamber is cylindrical in geometry of diameter 0.7 cm and total height 7.6 cm with hemispherical top. The cylindrical length of the central electrode is 7.25 cm and the hemispherical top has the daimeter of 0.7 cm. The bottom plate is made up of graphite which contains venting holes, insulators, and an aluminum guard ring. A thin wall hollow plastic rod is also fixed at the bottom of the chamber (stem) for ease of handling and mechanical support during experimental setups. The air cavity is almost surrounded by graphite with only a small amount of insulating material exposed at the bottom of the central electrode. The voltage current characteristic curve of the chamber was generated and 500 V was selected as nominal operating voltage for low‐activity ${\;}^{137}\text{Cs}$ sources. As far as we know, a large‐volume guarded graphite ionization chamber of such a design is not available commercially.

### B. $N_{K}$ by analytical calculation

Consider a gas (air) filled graphite wall ionization chamber of volume V, positioned in a medium of density ρ and irradiated with a broad parallel beam of ${\;}^{137}\text{Cs}$ gamma rays. The gas‐filled ionization chamber forms a cavity in the medium and the measured quantity is the ionization charge *Q* (C) from the mass $m_{g}$ (kg) of the gas. Mean absorbed dose (Gy) in the cavity gas is given by the relation:^(^ ^31^ ^)^

(1) $${\bar{D}}_{g}=\left(\frac{Q}{m_{g}}\right){\left(\frac{\bar{W}}{e}\right)}_{g}$$

where ${\left(\bar{W}/e\right)}_{g}$ is the mean energy required to produce an ion pair in the dry gas (air)=33.97 J/C.

For the $1000\;{\text{cm}}^{3}$ graphite wall air filled chamber, the ratio of mean absorbed dose in the cavity gas ${\bar{D}}_{g}$ and graphite (carbon) wall $D_{c}$ can be given by the Burlin's general cavity theory^(^ ^31^ ^)^ as:

(2) $$\frac{{\bar{D}}_{g}}{D_{c}}=d{\left(\frac{\bar{S}}{\rho }\right)}_{c}^{g}+(1-d){\left(\frac{{\bar{\mu }}_{en}}{\rho }\right)}_{c}^{g}$$

where ${\left(\bar{S}/\rho \right)}_{c}^{g}$ is the ratio of mean mass collision stopping power of the gas in the cavity and that of the carbon wall, ${\left({\bar{\mu }}_{en}/\rho \right)}_{c}^{g}$ is the ratio of mean mass energy absorption coefficient of the gas in the cavity and that of the carbon wall, *d* is the fraction of the dose due to electrons from the medium (Bragg‐Gray part), and (1−d) is the fraction of the dose from interactions in the cavity (large cavity/photon detector part).

Combining Eqs. (1) and (2), we get:

(3) $$D_{c}=\left(\frac{Q}{m_{g}}\right){\left(\frac{\bar{W}}{e}\right)}_{g}{\left[d{\left(\frac{\bar{S}}{\rho }\right)}_{c}^{g}+(1-d){\left(\frac{{\bar{\mu }}_{en}}{\rho }\right)}_{c}^{g}\right]}^{-1}$$

If the cavity is replaced by air, which is the medium of interest in measuring RAKR or AKS, the dose to air (medium) $D_{a}$ can be obtained using the relation:

(4) $$D_{a}=D_{c}{\left(\frac{{\bar{\mu }}_{en}}{\rho }\right)}_{c}^{a}A_{w}$$

where ${\left({\bar{\mu }}_{en}/\rho \right)}_{c}^{a}$ is the ratio of mean mass energy absorption coefficient of the air and that of the carbon wall, and $A_{w}$ is the wall correction factor which takes into account the chamber wall attenuation and scattering effects.

From Eqs. (3) and (4), we get:

(5) $$D_{a}=\left(\frac{Q}{m_{g}}\right){\left(\frac{\bar{W}}{e}\right)}_{g}{\left(\frac{{\bar{\mu }}_{en}}{\rho }\right)}_{c}^{a}{\left[d{\left(\frac{\bar{S}}{\rho }\right)}_{c}^{g}+(1-d){\left(\frac{{\bar{\mu }}_{en}}{\rho }\right)}_{c}^{g}\right]}^{-1}A_{w}$$

Under the condition of CPE, $D_{a}=K_{a,col}$ where $K_{a,\mathrm{col}}$ is the collision air kerma. Thus Eq. (5) can be written as:

(6) $$\frac{K_{a,col}}{Q}\left(\frac{1}{m_{g}}\right){\left(\frac{\bar{W}}{e}\right)}_{g}{\left(\frac{{\bar{\mu }}_{en}}{\rho }\right)}_{c}^{a}{\left[d{\left(\frac{\bar{S}}{\rho }\right)}_{c}^{g}+(1-d){\left(\frac{{\bar{\mu }}_{en}}{\rho }\right)}_{c}^{g}\right]}^{-1}A_{w}$$

The radiative component of the air kerma in the case of ${\;}^{137}\text{Cs}$ is < 0.1%; hence, $K_{a,\text{col}}=K_{a}$. Accordingly, the air kerma calibration coefficient $\left(N_{K}=K_{a}/Q\right)$ for the $1000\;{\text{cm}}^{3}$ cylindrical graphite ionization chamber can be given by:

(7) $$N_{K}=\left(\frac{1}{m_{g}}\right){\left(\frac{\bar{W}}{e}\right)}_{g}{\left(\frac{{\bar{\mu }}_{en}}{\rho }\right)}_{c}^{a}{\left[d{\left(\frac{\bar{S}}{\rho }\right)}_{c}^{g}+(1-d){\left(\frac{{\bar{\mu }}_{en}}{\rho }\right)}_{c}^{g}\right]}^{-1}A_{w}$$

Equation (7) is a fundamental analytical equation which can be used for calculating the air kerma calibration coefficient of the $1000\;{\text{cm}}^{3}$ ionization chamber.

The mass of the gas (air) $m_{g}=\rho_{0}V$, where $\rho_{0}=1.205\;\times 10^{-3}\;{\text{gcm}}^{-3}$ is the dry air density at the reference temperature and pressure (20°C and 101.325 kPa), and *V* is the cavity volume. The measured geometric parameters of the cylindrical cavity and the central electrode were used to determine cavity volume *V* and mass of the gas in the cavity. The value of parameter *d* was calculated using the following relations:^(^ ^31^ ^)^

(8) $$d=\frac{1-e^{-\beta L}}{\beta L}$$

(9) $$\beta =\frac{16\rho }{{\left(T_{\max}-0.036\right)}^{1.4}}$$

where L=4V/S (S is the surface area of the cavity) is the mean chord length, and β is the attenuation coefficient (cm^−1^), ρ is the gas density (gcm^−3^), and $T_{\max}$ is the maximum energy of the secondary electrons (MeV) produced by 662 keV ${\;}^{137}\text{Cs}$ gamma rays.

The chamber wall correction factor $A_{w}$ was calculated using the expression:^(^ ^31^ ^)^

(10) $$A_{w}=1-\rho_{c}t{\left(\frac{\mu_{en}}{\rho }\right)}_{c}$$

where $\rho_{c}$ is the density of graphite (carbon) wall, *t* is the wall thickness of the chamber, and ${\left(\mu_{en}/\rho \right)}_{c}$ is the mass energy absorption coefficient of the carbon wall. Table 1 shows the values of the parameters/coefficients used for calculating $N_{K}$ for the cylindrical graphite ionization chamber using Eq. (7).

**Table 1:** Values of parameters and coefficients used to calculate $N_{K}$ by analytical method for $1000\;{\text{cm}}^{3}$ cylindrical graphite ionization chamber at 662 keV gamma rays from ${\;}^{137}\text{Cs}$ brachytherapy source.

*Parameter/Coefficients*	*Numerical Value*
Air density $\rho_{0}(\text{at}\;20{}^{\circ}C\;\text{and}\;101.325\;{\text{kP}}_{a})$	$1.205\;\text{Kg}/m^{3}$
${\left(\bar{W}/e\right)}_{c}$	33.97 J/C
${\left({\bar{\mu }}_{en}/\rho \right)}_{c}^{g}$	1.0011
${\left(\bar{s}/\rho \right)}_{c}^{g}$	0.9901
d	0.8353
$A_{w}$	0.9794

### C. $N_{K}$ by Monte Carlo simulation

The Monte Carlo N‐Particle transport code (MCNP 4C2) was used to compute air kerma calibration coefficient of the $1000\;{\text{cm}}^{3}$ cylindrical graphite ionization chamber for ${\;}^{137}\text{Cs}$ low‐dose rate brachytherapy source. MCNP is a general purpose continuous energy, generalized geometry, time‐dependant code, which deals with transport of neutrons, photons, and coupled electron photon transport i.e., transport of secondary electrons resulting from gamma interactions.^(^ ^32^ ^)^ The photon physics option of the code utilized in this work includes coherent scattering, incoherent (Compton) scattering, photoelectric effect (with K and L shell fluorescence), and pair production.^(^ ^33^ ^)^ MCNP was run in the Mode: P with energy cutoff value of 1 keV to terminate particle transport.

An MCNP model, including the geometrical representation of the locations of the ionization chamber and Amersham CDCS‐J‐type ${\;}^{137}\text{Cs}$ source, was realized using the geometry input options available in the code. In the Monte Carlo simulation, the source and the chamber were housed inside a concrete walled room of dimensions 3.0 m×3.0 m×3.0 m to take into account the effect of Compton scattered photons. During the simulation, the ${\;}^{137}\text{Cs}$ source was located 120 cm above the ground and at 100 cm distance from the walls of concrete room in order to represent the actual experimental setup used during experimental measurement. A constant distance of 100 cm between the centers of the source and the chamber was maintained. Atomic composition (by weight) of the source materials used in the MC calculations is taken from the literature.^(^ ^34^ ^)^ Source self attenuation and the attenuation and scattering in the surrounding materials were considered in the Monte Carlo computations. The room was filled with air of density $1.205\times 10^{-3}\;{\text{gcm}}^{-3}$, and 50 cm thick concrete of density $2.35\;{\text{gcm}}^{-3}$ was assumed along the floor, ceiling, and side walls. Gamma energy of 662 keV of ${\;}^{137}\text{Cs}$ emission (yield of 662 keV: 0.851 photon/disintegration) was used during the simulation.^(^ ^35^ ^)^ The source is normalized to one Bq of ${\;}^{137}\text{Cs}$. In this study, the initial source weight denotes the number of photons starting from the source per unit decay of ${\;}^{137}\text{Cs}$ radionuclide.

The main aim of utilizing MCNP to simulate the material, geometry, and source details of the system is to predict the RAKR from the source and the calibration coefficient of the ionization chamber. The MCNP tally options utilized for this problem are the *F5 (photon energy fluence) and F6 (photon energy deposition) tallies with Mode: P, to evaluate the required parameters of interest viz. the photon energy fluence and energy deposition rates in the chamber. In *F5 tally, the radius of the sphere of exclusion of 1/8 to 1/2 mean free paths (a fixed value of 0.1 cm was used as exclusion distance) was maintained for particles of average energy at the sphere.^(^ ^36^ ^)^ The air kerma was estimated from the computed photon energy fluence at a distance of 1 m from the source using the mass energy absorption coefficient $\left(0.0293\;{\text{cm}}^{2}/g\right)$. It may be noted that the energy fluence computed by the *F5 tally of MCNP includes the effect of attenuation and scattering from the source matrix and all surrounding media. Here it is assumed that the radiative losses are negligible. Therefore, the collision and total air kerma are indistinguishable from each other. The air kerma per source photon is calculated from the estimated photon energy fluence (obtained from *F5 tally) by using the following expression:

(11) $$K_{a}\;(Gy/source\;photon)=1.602x10^{-10}\Psi (E){\left(\frac{\mu_{en}(E)}{\rho }\right)}_{a}$$

where *E* is the photon energy, Ψ(E) is the photon energy fluence $\left({\text{MeVcm}}^{-2}\right)$ at energy E at 1 m from the source, ${\left(\overset{\mu_{en}(E)}{\;}/\underset{\rho }{\;}\right)}_{a}$ is the mass energy absorption coefficient of air at energy E, and $1.602\times 10^{-10}$ is the factor to convert $K_{a}$ from MeV/g to Gy.

The energy deposition rate was estimated by using the F6 tally of the MCNP code. The energy deposition rate in the sensitive volume of the chamber obtained in units of ${\text{MeVg}}^{-1}$ per source photon from the MCNP code was converted to ionization chamber charge reading by using the following relation:

(12) $$\text{Charge}\;(C/source\;photon)=\frac{A(MeV/g)B(J/MeV)m(g)}{{\left(\bar{W}/e\right)}_{g}(J/C)}$$

where *A* is the energy deposition per unit mass in the sensitive volume of the chamber computed using the F6 tally, *B* is a conversion factor equal to $1.602\times 10^{-13}$ J/MeV, *m* is the mass of air enclosed in the chamber cavity, and ${\left(\bar{W}/e\right)}_{g}$ is equal to 33.97 (J/C).

### D. $N_{K}$ by experimental measurement

**Figure 2:**
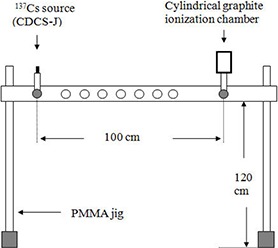
Schematic diagram of experimental setup used to determine the air kerma calibration coefficient of the cylindrical graphite ionization chamber.

The IAEA calibrated Amersham CDCS‐J‐type ${\;}^{137}\text{Cs}$ source was used in the experiment. The RAKR of this ${\;}^{137}\text{Cs}$ source on the day of measurement was $159.35\times 10^{-6}{\text{Gyh}}^{-1}$ with the reported uncertainty of less than 2% at 95% confidence level. To measure the charge rate using cylindrical ionization chamber experimentally, a specially designed perspex (PMMA) jig was fabricated capable of holding the CDCS‐J‐type ${\;}^{137}\text{Cs}$ source and $1000\;{\text{cm}}^{3}$ ionization chamber (Fig. 2). A constant separation of 100±0.2 cm between the centers of the source and chamber was maintained throughout the experimental measurements. The source center was aligned with the chamber center mechanically by adjusting the height of the chamber. During the experiment, the ionization chamber was irradiated continuously while electrometer readings were recorded at 15‐minute intervals. The average reading was corrected for variation in environmental conditions, ion‐recombination, and air attenuation. Nonuniformity correction factor, both for isotropic electron fluence in the air cavity (Kondo and Randolph^(^ ^37^ ^)^) as well as for anisotropic electron fluence in the air cavity (Bielajew^(^ ^38^ ^)^), was also determined and applied while calculating the corrected charge rate.^(^ ^13^ ^,^ ^37^ ^–^ ^39^ ^)^ Room scatter correction factor was determined by the shadow shield method and cross‐checked by the Monte Carlo method. The $N_{K}$ of the chamber was calculated using decay‐corrected RAKR of the source provided by the IAEA $\left({\mathrm{RAKR}}_{\mathrm{IAEA}}\right)$ and the corrected ionization chamber measured charge rate using the following expression:

(13) $${\text{NK}}_{,\text{expt}}(\text{GY}/C)={\text{RAKR}}_{\text{IAEA}}({\text{Gyh}}^{-1})/\text{Corrected}\;\text{charge}\;\text{rate}({\text{Ch}}^{-1})$$

## III. RESULTS & DISCUSSION

Three values of the air kerma calibration coefficients of the $1000\;{\text{cm}}^{3}$ cylindrical graphite ionization chamber – $N_{K}$ by analytical method $\left(N_{K,\text{analytical}}\right)$, $N_{K}$ by Monte Carlo method $\left(N_{K,\text{MC}}\right)$, and $N_{K}$ by experimental measurement $\left(N_{K,\text{expt}}\right)$ ‐ are $2.78\times 10^{4}(\text{Gy}/C),2.81\times 10^{4}(\text{Gy}/C)$, and $2.79\times 10^{4}(\text{Gy}/C)$, respectively. The percentage deviation between $N_{K,\text{analytical}}$ and $N_{K,\text{expt}}$ is 0.36%, and between $N_{K,\text{MC}}$ and $N_{K,\text{expt}}$ is 0.72%. The excellent comparison among the three $N_{K}$ values validates its analytically estimated value, and establishes this chamber as a reference standard for RAKR or AKS measurement of ${\;}^{137}\text{Cs}$ brachytherapy sources.

Table 2 presents the nonuniformity correction factor $\left(k_{n}\right)$ of the cylindrical graphite ionization chamber. The data includes nonuniformity correction factor as noted in the isotropic theory of Kondo and Randolph, as well as the nonuniformity correction factor according to the anisotropic theory of Bielajew. The nonuniformity correction factor determined as per Bielajew was applied during the calculation of corrected charge rate because this theory is an extension of the Kondo and Randolph theory, and includes a more realistic angular distribution of electron fluence in the air cavity of the chamber.

**Table 2:** Nonuniformity correction factors for the $1000\;{\text{cm}}^{3}$ cylindrical graphite ionization chamber.

	*Non‐uniformity Correction Factor as per:*	
*Measurement Distance (cm)*	*Kondo and Randolph* ^(^ ^37^ ^)^	*Bielajew* ^(^ ^38^ ^,^ ^39^ ^)^
10	0.9935	1.0203
20	0.9971	1.0101
30	0.9985	1.0061
40	0.9990	1.0047
50	0.9994	1.0036
60	0.9996	1.0029
80	0.9994	1.0022
100	0.9998	1.0019

The combined standard uncertainty $\left(u_{c}\right)$ in the estimation of the calibration coefficient of the graphite cylindrical ionization chamber by the analytical method (which is the quadrature sum of Type A and Type B uncertainties of the parameters used for calculating $N_{K}$), was estimated to be 1.1% (k=1). The uncertainty (Type A) due to the statistical fluctuations in the MCNP estimate of the $N_{K}$ is less than 0.01% (k=1) for $2\times 10^{8}$ particle histories considered in the simulations. This uncertainty estimate does not include the Type B uncertainties in the compiled photon cross sections, reported to be about 1% in the energy range of interest for low Z materials like graphite.

In the experimental determination of $N_{K}$, the combined standard uncertainty was calculated using the Type A and Type B uncertainties of the parameters including all possible components of uncertainty, as shown in Table 3.^(^ ^40^ ^)^ The $u_{c}$ works out to be 1.15% (k=1). This value of $u_{c}$ was multiplied by a coverage factor k=2 to obtain the expanded uncertainty (U) in $N_{K}$. The expanded uncertainty has a confidence probability of 95%, calculated from the effective number of degrees of freedom, $\left(v_{\text{eff},26}\right)$. The expanded uncertainty in the experimental determination of $N_{K}$ is 2.3% at 95% confidence limit (k=2).

**Table 3:** Uncertainty in experimental determination of air kerma calibration coefficient $\left(N_{K}\right)$ of the cylindrical graphite ionization chamber.

	*Relative Standard Uncertainty (%)*	
*Uncertainty Components*	*Type A*	*Type B*
Charge collection reproducibility	0.1	
Electrometer calibration		0.2
Leakage (Chamber +electrometer)		0.05
Temperature pressure correction factor (ktp)	0.2	
Timer accuracy	0.1	
Positional accuracy and reproducibility	0.2	
Room scatter correction factor (ksc)	0.12	
Ion recombination correction factor (krecom)		0.04
Nonuniformity correction factor (kn)		0.4
Air attenuation correction factor (katt)		0.1
RAKR of the 137Cs source		1.0
Combined standard uncertainty (k=1)	1.15 %	
Expanded uncertainty (k=2)	2.3 %	

The $N_{K}$ value computed by the MCNP Monte Carlo code includes the effect of air attenuation and scattering from the source and surrounding materials. The overall uncertainty in the Monte Carlo simulation consists of two parts: (i) the uncertainties in representation of the finer geometrical and physical details of the system (uncertainty up to 1% is expected in the compiled photon cross‐section data), and (ii) the inherent statistical fluctuations of the Monte Carlo method. The former can be improved by describing the finer physics and geometrical details of the system as faithfully as possible in the code input. The code will not be able to debug any input error or mismatch between the input values and the actual physical system under consideration. The latter part viz. the precision of the simulation, however, may be improved upon by ensuring adequacy in sampling of photon and electron histories. In analogue mode, this may be achieved by increasing the number of histories. The value of statistical precision is quoted as the relative standard error (i.e., the fractional standard deviation) of the simulated value. From the above discussion, we may infer that if the details of the system are accurately modeled in the input, one can ensure that the overall uncertainty of the simulation is reasonably small by sampling an adequate number of particle history tracks.

At photon energies higher than 300 keV, the greater range in air of the secondary electrons restricts the practical use of free air ionization chambers. For dealing with the calibration of photon‐emitting brachytherapy sources of energy higher than 300 keV, cavity ionization chambers are preferred because they are easier to use and have relatively lower uncertainties.^(^ ^29^ ^)^ NIST uses a large volume (2.8 L) spherical cavity ionization chamber made of an aluminum alloy for calibration of LDR ${\;}^{137}\text{Cs}$ brachytherapy sources.^(^ ^30^ ^)^ The cylindrical graphite ionization chamber described in this work was fabricated and established as a reference standard to calibrate the dosimeters of the hospitals, and are routinely used for strength measurements of ${\;}^{137}\text{Cs}$ brachytherapy sources.

## IV. CONCLUSIONS

A guarded cylindrical graphite ionization chamber of nominal volume $1000\;{\text{cm}}^{3}$ was designed and fabricated locally for use as a reference standard for ${\;}^{137}\text{Cs}$ brachytherapy sources. The air kerma calibration coefficient of this ionization chamber for ${\;}^{137}\text{Cs}$ source was determined by analytical calculations, by Monte Carlo simulation, and also by using a reference ${\;}^{137}\text{Cs}$ source. The three values of the $N_{K}$ of this chamber determined by three independent methods were found in excellent agreement. This ionization chamber can now be used to standardize low‐dose rate ${\;}^{137}\text{Cs}$ brachytherapy sources, and can also be used to calibrate hospital dosimeters.

## ACKNOWLEDGMENTS

The authors wish to express their gratitude to Dr. A. K. Ghosh, Director, Health Safety and Environment Group (HS&EG), Bhabha Atomic Research Centre (BARC), Dr. D. N. Sharma, Associate Director, HS&EG & Head, Radiation Safety Systems Division, BARC, and Dr. Y. S. Mayya, Head, Radiological Physics & Advisory Division (RP&AD), BARC, Mumbai, India for their encouragement and support during this work.
